# Identifying Reservoirs of Infection: A Conceptual and Practical Challenge

**DOI:** 10.3201/eid0812.010317

**Published:** 2002-12

**Affiliations:** * University of Edinburgh, Roslin, U.K.

**Keywords:** reservoir, epidemiology, pathogen, disease control, transmission

## Abstract

Many infectious agents, especially those that cause emerging diseases, infect more than one host species. Managing reservoirs of multihost pathogens often plays a crucial role in effective disease control. However, reservoirs remain variously and loosely defined. We propose that reservoirs can only be understood with reference to defined target populations. Therefore, we define a reservoir as one or more epidemiologically connected populations or environments in which the pathogen can be permanently maintained and from which infection is transmitted to the defined target population. Existence of a reservoir is confirmed when infection within the target population cannot be sustained after all transmission between target and nontarget populations has been eliminated. When disease can be controlled solely by interventions within target populations, little knowledge of potentially complex reservoir infection dynamics is necessary for effective control. We discuss the practical value of different approaches that may be used to identify reservoirs in the field.

Infectious agents that can infect more than one host species are ubiquitous. Indeed, 62% of all human pathogens are classified as zoonoses (1), and 77% of livestock pathogens and 91% of domestic carnivore pathogens infect multiple hosts. Fifty seven of the 70 animal diseases considered to be of greatest international importance infect multiple hosts (2). The ability of pathogens to infect a wide range of hosts has been demonstrated as a risk factor for disease emergence in both humans (1) and domestic animals (2). (Virtually all recent outbreaks of disease in endangered wildlife have been caused by pathogens that can infect other, more abundant host species [3,4]).

Pathogens that infect more that one host species are by definition likely to be encountered in several host populations, some of which may constitute infection reservoirs. Therefore, a key issue in the design of control measures for multihost pathogens is defining what is meant by reservoirs of infection and developing guidelines for their identification.

Although many emerging diseases of human, domestic animal, and wildlife populations are assumed to be maintained in reservoir hosts (4), these reservoirs are rarely identified. In recent years, several emerging infectious disease threats to human and animal health have been managed through large-scale measures directed at suspected reservoirs of infection. Sometimes action arises from a clearly perceived notion of where infection resides. For example, approximately 1 million pigs were slaughtered in Malaysia in 1999 to control the Nipah virus (5); several million chickens were slaughtered in Hong Kong in 1998 and 2001 to prevent a projected pandemic of *Influenza A virus* (6); and several million cows were slaughtered in Britain to curtail the epidemic of bovine spongiform encephalopathy**,** and its possible transmission to humans (7). However, many situations exist in which the role of reservoirs is less clear; for example, the reservoirs that harbor emerging viruses such as Ebola and Marburg remain unknown. For *Mycobacterium bovis* in the United Kingdom, a complex reservoir system seems most likely, and identification of the most important source of infection for cattle remains highly controversial (8). Incomplete understanding of reservoirs has hampered control of many diseases in Africa, such as Ebola virus infection, Buruli ulcer, and rabies (9–13).

Many different and often contradictory definitions of reservoirs exist. Studies stress different characteristics of reservoirs, namely, that infections in reservoir hosts are always nonpathogenic; any natural host is a reservoir host; the reservoir must be a different species; reservoirs are economically unimportant hosts; or reservoirs may be primary or secondary hosts (14–18). Some definitions imply that a reservoir comprises only one species; other definitions suggest that an ecologic system may act as a reservoir (16,18). Confusing, conflicting, and often incomplete concepts of what constitutes a disease reservoir result. We propose a conceptual framework for defining and identifying reservoirs and discuss the practical value of different approaches that may be used to identify reservoirs in the field.

## Proposed Framework

**Figure 1:**
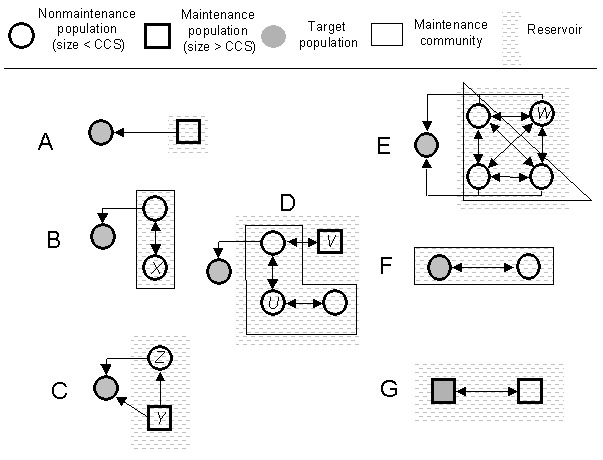
Examples of simple and more complex target-reservoir systems. In the simplest case, A, a maintenance population transmits a pathogen (indicated by arrows) to a target population that is smaller than the critical community size (CCS) and therefore classified as nonmaintenance. In B, the reservoir is composed of two connected nonmaintenance populations, only one of which is the source population, and neither of which could constitute a reservoir alone (typically akin to some vector-borne infections). Elimination of infection in population X will result in elimination of infection in the target. C depicts a situation in which Y is a maintenance population, but transmission can occur directly between Y and the target population or through another source population, Z. Although not essential to pathogen maintenance, Z is still part of the reservoir because it contributes to transmission of the pathogen to the target. In D, four nontarget populations must be included within the reservoir if its full dynamics are to be understood. Elimination of infection in U will not result in elimination of infection in the target, as V is an independent maintenance population. In E, all populations are sources. F illustrates that the target population itself may constitute part of the reservoir and G that the target population can be a maintenance population. If W is not required to maintain the infection, then W falls outside the maintenance community but is still part of the reservoir because it is a source.

We propose the following approach, which can be applied to any disease system, for understanding the role of potentially relevant reservoirs in that system. Figure 1 illustrates how this framework might be applied to various systems.

### Suggested Terminology

The target population is the population of concern or interest to us. All other potentially susceptible host populations that are epidemiologically connected directly or indirectly to the target population are nontarget populations and could potentially constitute all or part of the reservoir. If we are interested in protecting humans (the target species) from cryptosporidiosis, for example, the wide range of domestic and wild animal species in the environment in which *Cryptosporidim parvum* occurs (19) is the nontarget population, and those species constitute potential reservoir hosts.

In epidemiologic theory, the critical community size is the minimum size of a closed population within which a pathogen can persist indefinitely (20). In smaller populations the number or density of infected hosts frequently falls to low levels, random extinction (fadeout) becomes inevitable, and the pathogen cannot persist. Populations smaller than the critical community size, or those rendered effectively smaller than that critical size through control measures, we term nonmaintenance populations. Pathogens will persist in populations larger than the critical community size, and these populations we term maintenance populations. In complex systems, pathogen transmission between a number of nonmaintenance populations could constitute a maintenance community. Any population that transmits infection directly to the target population, we defined as a source population. Source populations may themselves be maintenance populations or, alternatively, may constitute all or part of a transmission link from a maintenance population to the target population.

If a target population is smaller than the critical community size and thus cannot maintain a pathogen, completely isolating the target population from any transmission from outside (ring-fencing) will cause the pathogen to become extinct in the target population. A reservoir is present if the pathogen repeatedly appears in such a nonmaintenance target population. For example, completely preventing tick transmission of *Borrelia* spirochetes to humans from other species would result in Lyme disease’s disappearance from humans; thus, a reservoir must exist. This procedure for identifying reservoirs will not apply to maintenance target populations. However, in practical terms reservoirs generally only become of concern when disease control within the target population reduces transmission within a target population to a very low level relative to transmission from nontarget to target populations. For example, *Foot-and-mouth disease virus* (FMDV) is maintained in unvaccinated cattle populations in many parts of Africa. The identification of wildlife reservoirs (e.g., buffalo) generally only becomes important once vaccinated cattle can no longer maintain infection at the population level, as is the case, for example, in parts of southern Africa (21).

We propose that a reservoir be defined as one or more epidemiologically connected populations or environments in which the pathogen can be permanently maintained and from which infection is transmitted to the defined target population. Populations in a reservoir may be the same or a different species as the target and may include vector species. As long as a reservoir constitutes a maintenance community and all populations within the maintenance community are directly or indirectly connected to each other, the size of the reservoir has no upper limit.

### Previous Concepts of Reservoirs

Previous reservoir definitions often required that the relevant infectious agent be nonpathogenic to the reservoir host species (14,15). However, pathogenicity, per se, has little bearing on the persistence of infectious agents in populations. Excluding the possibility of a reservoir solely because the infectious agent was pathogenic to a nontarget host—as is the case with pathogens such as Nipah, Hendra, and rabies viruses and with bovine spongiform encephalopathy**—**would clearly be a mistake.

Cleaveland and Dye (12) proposed criteria to identify reservoir hosts but did not take into account multihost aspects of reservoirs. Swinton et al. (16) used the terms reservoir and satellite to describe the dynamics of *Phocine distemper virus* in the North Sea population of harbor seals (*Phoca vitulina*). Infection from a satellite population effectively induces persistence of infection in the reservoir population (17). Neither population constitutes a maintenance population, but infection can be maintained in a coupled system (illustrated in Figure 1B). Both satellite and reservoir populations would be components of our reservoir.

In an insightful paper, Ashford recognized many of the problems in the simplistic use of the term reservoir and proposed a consistent definition of a reservoir as an “ecological system in which the infectious agent survives indefinitely” (18). This definition differs from ours in that it does not reference a target population and thus does not require that a reservoir be a source of infection for a target population. Ashford defined reservoir hosts as those essential to maintenance of the pathogen. We, however, argue that reservoirs may include nonessential hosts. Excluding nonessential hosts from a reservoir causes two problems. First, populations harboring infection may be nonessential to maintenance yet play a major role in transmitting the pathogen to the target population. For example, FMDV persists indefinitely in African buffalo herds; yet impala may constitute an important source of infection for the cattle target population (22) (e.g., population Z in Figure 1C). Second, as Ashford recognized, the definition of reservoir membership becomes ultimately intractable if each constituent population in the reservoir is considered nonmaintenance. Under these circumstances, a reservoir could be composed of subsets of nonmaintenance populations in a variety of ways (Figure 1D). Although a minimal definition of a reservoir is clear, a fully inclusive definition is much less so. In Figure 1E, population V is not an essential host; nonetheless, this population must be considered a component of the reservoir because, if infection is eliminated in the rest of the reservoir, eradication would not be achieved. For the same reason, our concept of a reservoir differs from the notion of a critical species assemblage, which is defined as the minimum set of host communities in which a parasite can persist (16).

## Control of Infection

Practical disease control requires answers to two questions: 1) Can an acceptable level of control be accomplished without consideration of a reservoir? 2) If not, what populations constitute the reservoir? Given a target-reservoir system, policies to manage infection may contain elements of three broadly different tactics: 1) target control: directing efforts within the target population with no reference to the reservoir (e.g., human vaccination against yellow fever [23]); 2) blocking tactics: directing control efforts at blocking transmission between source and target populations (e.g., game fences to control FMDV in cattle); and 3) reservoir control: controlling infection within the reservoir (e.g., culling programs, vaccination, or treatment of reservoirs). These three approaches require progressively increased levels of understanding of reservoir structure and function.

Target control has the important advantage of requiring no knowledge of potentially complex reservoir dynamics. A complete understanding of infection dynamics within the reservoir is also not necessary to implement blocking tactics, although identifying source populations in the reservoir is essential. The more precisely that source populations can be identified and the more quantitative data that are available on their relative contribution to transmission, the more efficient the allocation of resources is for disease control. Reservoir control tactics require a much more complete understanding of the structure and transmission processes that occur within the reservoir. For example, efforts directed at controlling infection in nonmaintenance components of a reservoir are unlikely to be effective if infection in the maintenance component of the reservoir remains uncontrolled.

**Figure 2:**
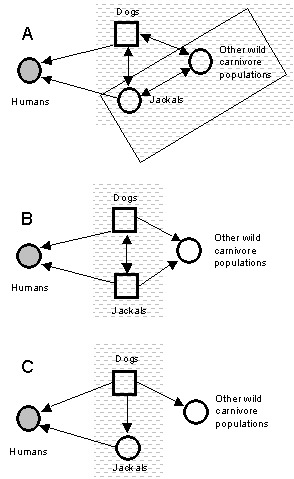
Potential complexity of rabies reservoirs in Zimbabwe. If jackals with (A) or without (B) other wild carnivore populations constitute a maintenance community independent of dogs, then vaccination of dogs alone will not result in rabies elimination in the target. If jackals do not constitute a maintenance community independent of dogs (C), then dog vaccination should clear rabies from the reservoir (symbols as in Figure 1).

The practical problem of identifying reservoirs of rabies for humans in Zimbabwe provides a useful illustration of some issues involved. After a rise in the incidence of jackal and dog rabies in the 1990s, debate has centered on whether jackals (*Canis adustus*) are reservoirs of this disease, an issue that has important implications for formulating national rabies-control programs (10,11). In Zimbabwe, domestic dogs are a maintenance and source population of rabies for humans. However, jackals account for >25% of all confirmed rabies cases in animals and are also an important source of infection for humans (10,11). Jackals may be important components of the reservoir as a maintenance or nonmaintenance population (Figure 2). Because rabies can be maintained in dogs without jackals, jackals are not an essential constituent population of the reservoir. But can infection persist in jackals without dogs (Figure 2B)? Jackals may constitute part of a maintenance community in conjunction with an assemblage of other wild carnivores (Figure 2A). The question is important because if dogs are the only maintenance population in the reservoir, effective vaccination campaigns targeted at dogs should successfully eliminate human rabies from Zimbabwe. If, however, jackals comprise all or part of a maintenance community independent of dogs, eliminating rabies will only be successful if jackal rabies were also controlled (10,11). The recent high incidence of jackal rabies in Zimbabwe might suggest that jackals are maintenance populations. A high incidence of disease alone is neither necessary nor sufficient evidence for this claim, particularly when wide fluctuations in disease incidence occur (as with jackal rabies). Mathematical models suggest that jackals are probably unable to support infection without frequent reintroductions from outside sources (24). However, Bingham et al. (11) argue that spatial patterns are critical and that jackal epidemics may be sustained independently within key geographic areas. The issue can be resolved unequivocally through implementation of a mass dog vaccination campaign, which would be a logical first phase of a national program. If jackal rabies persists in the absence of dog rabies, an effective program for rabies elimination will likely need to include oral vaccination of jackals.

Rabies also provides an example of the need to identify a target population when defining reservoirs. In the Serengeti Plain in Tanzania, a distinct strain of rabies appears to be maintained independently in spotted hyenas, without causing them any clinical disease, and with no evidence of spillover infection or disease occurring in any other species (within the limits of current knowledge) (25). By our definition, unless this strain is identified as the cause of disease in another species (i.e., a target population), hyenas in the Serengeti cannot be considered as a reservoir of rabies.

## Practical Indicators To Identify Reservoirs

Newly emerging diseases usually originate from reservoirs of infection in other host species. When such diseases first appear, only rapid, accurate identification of the reservoir will enable appraisal of the full range of disease-control options. Ring-fencing is clearly impractical when no knowledge of the reservoir populations exists, but other steps can be taken to acquire progressively more detailed information about the reservoir structure.

### Epidemiologic Evidence of Association

Accumulating epidemiologic evidence is often the best first step in identifying a reservoir. Initially, such analyses are often based on sparse data and are rarely published. Links between target and reservoir may be particularly elusive when transmission from reservoir to target is rare or sporadic, as, for example, occurs with Ebola virus or *Marburg virus* (26).

Quantitative data on risk factors for infection can be obtained through more formal epidemiologic research, such as case-control and cohort studies. For example, a case-control study of Borna disease in cats indicated that hunting mice was a risk factor and that rodents might be virus reservoirs (27). Case-control studies have identified badgers as risk factors for *M. bovis* infection in cattle in some parts of the United Kingdom (28). In other cases, putative reservoirs have been ruled out. For example, a risk factor analysis of *Helicobacter pylori* infection in young children showed that household pets were not incriminated (29). Although such associations may suggest a link between reservoir and target populations, further evidence is required to establish the identity of a reservoir.

### Evidence of Natural Infection in Nontarget Populations

Identifying natural infection is a useful step towards determining natural hosts that may constitute potential reservoirs. Natural infection may be determined in two ways: by identifying previous infection through antibody detection or by identifying current infection through isolating the infectious agent or its genes from the host. The appropriate approach depends on the longevity of the infection in the host and the resources available. For example, very large sample sizes might be required to isolate a virus from a reservoir population; a serologic survey might be less expensive and more feasible. In a number of studies, demonstration of natural infection has been considered strong evidence that hosts are reservoirs, e.g., *Leishmania* in small mammals in Iran (30) and hantavirus in rodents in the Americas (31).

Seropositivity indicates that infection has occurred. However, not all natural hosts are reservoir hosts, and to include a nontarget population in a reservoir, evidence of transmission to the target population, direct or indirect, must exist. Furthermore, the level of seroprevalence does not provide information as to whether a nontarget population is a maintenance host. High seroprevalence at a single point in time may simply indicate an outbreak in the host population, rather than pathogen persistence (32). Low seroprevalence may arise when case-mortality rates are high in the reservoir (as in rabies infections), during an interepidemic trough, or when a pathogen persists at a stable but low prevalence, particularly when the duration of the infectious period is high (e.g., as in carrier animals). The critical issue is the persistence of infection in the reservoir, which can only be determined through longitudinal studies.

Similar guidelines apply to data based on demonstration of the pathogen within a host. For example, detection of *Trypanosoma brucei gambiense* in wild ruminants and primates in West Africa has been taken as evidence of an animal reservoir for Gambian sleeping sickness (33). However, as animal-to-human transmission has never been demonstrated, wildlife remain classified as potential reservoir hosts, and disease control relies on treatment of people. In contrast, for Rhodesian sleeping sickness, isolation of *T. brucei rhodesiense* from a single bushbuck in the 1950s (34) led to the assumption that wildlife was the principal reservoir for human disease and resulted in widespread culling of wildlife for disease control. Only in 1966 were cattle identified as reservoir hosts (35). Current strategies focus on treating cattle with trypanocidal drugs (36).

Detecting a pathogen, particularly its transmission stage, in secretions or tissues provides supportive, but not unequivocal, evidence that transmission to the target population can occur. Even where experiments demonstrate that transmission is possible, it may not occur in nature for a variety of behavioral or social reasons, because the population is below critical community size or because of constraints of pathogen life history.

### Genetic/Antigenic Characteristics

Genetic and antigenic characterization of pathogens isolated from different populations provides a more powerful tool for identifying key components of reservoirs. Antigenic and genetic variation of pathogens isolated from the target population within the range observed in the reservoir is consistent with reservoir-target transmission**.** This pattern can be demonstrated by applying phylogenetic methods to sequence, random amplified polymorphic DNA, or restriction fragment length polymorphism data, or by using serum cross-reactivity studies. Such methods have also been used to rule out important animal reservoirs of human disease in studies of *Ascaris* in Guatemala (37) and *Cryptosporidium* in Australia (38).

### Intervention Studies

Complete ring-fencing of target populations is the ultimate step in identifying the existence and structure of reservoirs. In practice, however, ring-fencing has rarely been achieved and, as a result, even those reservoirs we consider to be most fully understood are not usually inconvertibly proven. Despite this, once a potential reservoir is identified, intervention studies can permit incidental but powerful inferences about the dynamics of infection in target-reservoir systems. In many cases, disease-control programs can effectively act as intervention studies.

Control in a reservoir host population may be achieved by reducing host or vector density (e.g., culling possums to control tuberculosis in New Zealand [39], mosquito control for West Nile fever, or sandfly control for cutaneous leishmaniasis [40]). Alternatively, control measures may focus more directly on preventing transmission from the reservoir, e.g., separation of cattle and wildebeest to prevent transmission of malignant catarrhal fever in East Africa (41). The success of such interventions often provides reasonable confirmation of the original assumptions concerning transmission and maintenance of infection in the target-reservoir system.

## Conclusions

We have a poorer understanding of the epidemiology of multihost pathogens than simpler single-host systems. This dearth of understanding is a particular problem with emerging diseases, since most emerging human, domestic animal, and wildlife diseases infect multiple hosts. Reservoirs must be defined with reference to particular target populations. Disappearance of the pathogen in the target population after ring-fencing provides categorical evidence of the existence of a reservoir and its possible identity. However, exhaustive identification of all constituent populations of a reservoir may be difficult. This identification need not be a management priority if disease control is directed at the target population or at blocking transmission between reservoir and target. For infection to be eliminated, however, disease-control measures must be directed at the reservoir. Thus, an understanding of reservoir infection dynamics is essential.

When the risks and costs of control are low, circumstantial evidence may be sufficient to justify implementing control measures. Specifically designed intervention studies have ultimately been required to determine whether a particular species is a maintenance host, a source of infection, or one that has been infected incidentally. Control measures are likely to be ineffective if they are directed at components of the reservoir that are neither maintenance hosts nor transmitters of the pathogen to the target population.
